# Asymmetric analogous hyperbola model of overburden movement and its verification

**DOI:** 10.1038/s41598-024-66622-9

**Published:** 2024-07-06

**Authors:** Liu Jiashun, Wang Yang, Zuo Jianping, Wu Zuoqi, Sun Yunjiang, Zheng Zhiyong

**Affiliations:** 1 School of Civil Engineering, Liaoning Technical University, Fuxin, 123000 Liaoning China; 2 School of Mechanics and Civil Engineering, China University of Mining and Technology-Beijing, Beijing, 100083 China; 3 China Coal Science and Technology Ecological Environment Technology Co., Ltd., Beijing, 100013 China

**Keywords:** Thick loose layer, Overburden rock, Asymmetric analogous hyperbola model, Similar model test, UDEC, Engineering, Civil engineering

## Abstract

The extraction of underground coal resources induces the fracture and movement of overlying strata, leading to geological hazards such as surface deformation, cracks, and even subsidence. Utilizing the analogous hyperbola model of overlying strata movement, we conducted a mechanical analysis to examine the asymmetric fracture mechanism resulting from coal seam mining in thick loose strata. An asymmetric analogous hyperbola model was established by introducing distinct virtual half-axis lengths (*b*). The thickness impact of thick loose layers (*H*) and bedrock layer (*h*) on the asymmetric movement of overlying rock during mining was also discussed. Similarity model tests were conducted to research the migration characteristics and surface subsidence patterns of overburdened rock and thick loose layers at different mining stages and validate the hypothesis of asymmetric overburdened rock migration. Additionally, the discrete element numerical model for thick and loose layers mining was established by using UDEC and discussed the asymmetric analogous hyperbola behaviour of overburden movement and surface subsidence. The comparison results show that the established asymmetric hyperbolic model can effectively predict the movement law of overlying strata and surface subsidence characteristics. Therefore, the proposed model can provide valuable theoretical support for predicting the movement patterns of overburden under thick loose layers and mitigating surface subsidence disasters.

## Introduction

The thickness loose layer denotes quaternary sediments with a soil thickness exceeding 50 m, which are widely distributed in China, such as North China, Northeast, and Northwest. The primary coal production centers in China are now shifting towards Shanxi, Shaanxi, Inner Mongolia, and Xinjiang. However, there is a noticeable disparity in coal production between the central and western regions in comparison to the eastern regions. The majority of coal mines in China utilize underground mining methods, which expose them to various risks of disasters^1–4^. Thick loose layers mining intensifies the displacement of rock strata and surface subsidence, resulting in safety and environmental concerns, including face bursts, extensive surface collapses, and soil erosion^5–7^. Therefore, an investigation into the movement characteristics of overlying strata beneath thick loose layers and precise prediction of surface subsidence regarding depth and extent are crucial for ensuring mining safety and averting surface subsidence disasters.

In thick loose layer mining, the movement of overlying rock is categorized by the key stratum^8,9^. A trapezoidal fracture exists below this layer, while there is a “funnel-shaped” sliding movement extending to the surface above it and this lead to a subsidence basin in the surface. This is also notably distinct from the trapezoidal fractures formed during thin or no loose layer mining^10–13^. Traditionally, rock movement and surface subsidence have been studied independently. Research on rock movement primarily delves into the mechanics of rock fracturing induced by mining activities, while investigations on surface subsidence centre around numerical values of surface collapse, subsidence range, and their implications for surface structures and the environment^14^. To tackle challenges related to overlying rock movement and surface subsidence induced by mining disturbances, Qian et al.^15,16^ first introduced the “block beam” model for rock movement, significantly advancing the foundational theoretical research in the field of mining in China. Xu et al.^17^ investigated the correlation between the key stratum in the rock stratum and surface subsidence, contributing to the advancement of coal mining research in China. Litwiniszyn^18^ incorporated the theory of random media into predicting surface subsidence resulting from mining activities. Following this, Liu et al.^19^ devised the probabilistic integral method for predicting surface movement and deformation in mining engineering. Jia et al.^20^ investigated the condition of direct roof fractures and introduced the elastic thin plate failure theory for rock strata. Jiang et al.^21^ proposed structural forms for rock strata roofs, including arches, arch beams, and beam structures, offering a theoretical foundation for dynamic pressure control. After coal seam mining, the collapse movement of overlying strata will directly affect the surface soil layer, causing disasters such as strong subsidence and surface collapse in the mining area. Through physical simulation experiments and theoretical analysis, the formation and development laws of surface cracks penetrating thick sandy soil layers are revealed^22^. Ma et al.^23^ divided the overlying strata into bedrock and loose layers, applying rheological theory and the Weibull subsidence equation to derive a composite model suitable for thick bedrock and thin loose layers. Zuo et al.^24–27^ integrated rock movement with surface loose layer subsidence, developing a series of analogous hyperbola model models for comprehensive rock movement. This significantly advanced the unified theoretical research on overlying rock movement and surface subsidence, leading to effective practical applications.

Substantial engineering evidence indicates that in thick coal seam mining, the initial collapse angle of the bedrock exceeds the periodic collapse angle. Bedrock fracturing and displacement during underground resource extraction show asymmetry, resulting in surface subsidence that deviates from the mining centre and exhibits non-central symmetric settling. Building upon this, an asymmetric analogous hyperbola model for overburden movement in thick loose layers was developed. This model takes into account the asymmetric bedrock collapse caused by varying collapse angles and introduces different virtual semi-axis lengths, denoted as *b*. Two-dimensional similar material model experiments and UDEC numerical simulations were conducted according to the engineering conditions of S12002 working face in Ningtiaota mine. The goal was to explore the patterns of asymmetric overburden movement and surface subsidence under thick loose layer mining conditions and to verify the reliability of the asymmetric analogous hyperbola model. The research findings will offer theoretical support for predicting overlying strata fracture movement and surface subsidence, as well as for disaster prevention and control, in the context of thick and loose layer mining.

## Mechanism of asymmetric fracture mechanics in mining rock strata

**Figure 1:**
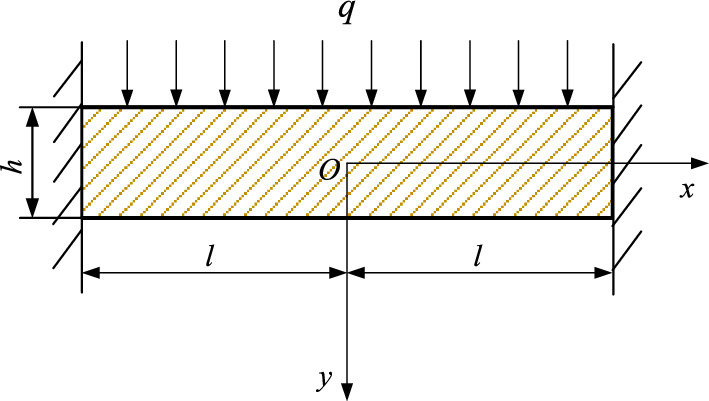
Initial fracture mechanics model of bedrock.

Yu et al.^28^ conducted empirical measurements and simulations to analyze surface subsidence resulting from coal mining operations. They identified the asymmetric characteristics of the initial fracture in the rock layer above the goaf. They explained that the differential collapse lengths at critical layers between the open-cut side and the final mining line contribute to asymmetric surface subsidence. Therefore, it is reasonable to infer that rock fracture and strata movement induced by coal mining are asymmetric. Focusing on the lowest rock stratum during the collapse of overlying strata, as the working face advances, it can be modeled as a fixed-end beam structure subjected to uniformly distributed loads, as illustrated in Fig. 1.

Utilising elasticity mechanics for analysis, the expression of stress components within a fixed beam is revealed^17^.

1 $$ \left\{ {\begin{array}{*{20}l} {\sigma_{x} = \frac{6qy}{{h^{3} }}\left( {l^{2} - x^{2} } \right) + \frac{qy}{h}\left( {\frac{{4y^{2} }}{{h^{2} }} - \frac{3}{5}} \right)} \hfill \\ {\sigma_{y} = - \frac{q}{2}\left( {1 + \frac{y}{h}} \right)\left( {1 - \frac{2y}{h}} \right)^{2} } \hfill \\ {\tau_{xy} = - \frac{6qx}{{h^{3} }}\left( {\frac{{h^{2} }}{4} - y^{2} } \right)} \hfill \\ \end{array} } \right. $$

The load *q* supported by any layer in the bedrock can be calculated using Eq. (2).

2 $$ \left( {q_{n} } \right)_{1} = \frac{{E_{1} h_{1}^{3} \left( {\gamma_{1} h_{1} + \gamma_{2} h_{2} + \gamma_{3} h_{3} + \cdots + \gamma_{{\text{n}}} h_{{\text{n}}} } \right)}}{{E_{1} h_{1}^{3} + E_{2} h_{2}^{3} + E_{3} h_{3}^{3} + \cdots + E_{{\text{n}}} h_{{\text{n}}}^{3} }} $$

where *E*_n_ is the elastic modulus of the *n*th rock layer, MPa. *h*_*n*_ is the thickness of the *n*th rock layer, m. *γ*_*n*_ is the bulk weight of the nth rock layer, kN/m^3^.

**Figure 2:**
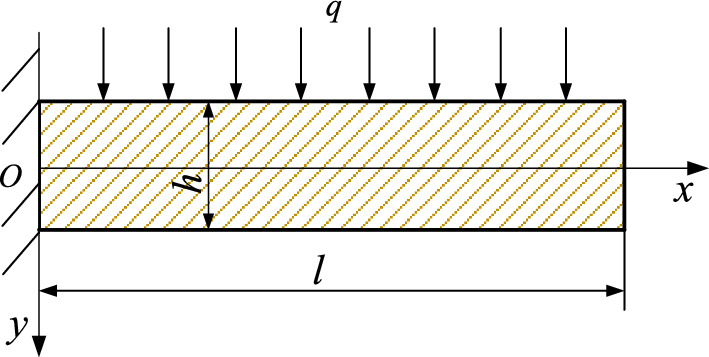
Periodic fracture mechanics model of bedrock.

With the progression of the working face, the bedrock initially experiences fractures followed by periodic fractures. This scenario can be conceptualized as a cantilever beam model subjected to a uniformly distributed load, as depicted in Fig. 2.

In elasticity mechanics, the expression for the stress components of a cantilever beam is as follows,

3 $$ \left\{ {\begin{array}{*{20}l} {\sigma_{x} = - \frac{6q}{{h^{2} }}x^{2} y + \frac{qy}{h}\left( {\frac{{4y^{2} }}{{h^{2} }} - \frac{3}{5}} \right)} \hfill \\ {\sigma_{y} = - \frac{q}{2}\left( {1 - \frac{3y}{h} + \frac{{4y^{3} }}{{h^{3} }}} \right)} \hfill \\ {\tau_{xy} = - \frac{6qx}{{h^{3} }}\left( {\frac{{h^{2} }}{4} - y^{2} } \right)} \hfill \\ \end{array} } \right. $$

The expression for the collapse angle *θ* after bedrock fracture is given by,

4 $$ \theta = \frac{\pi }{2} - \frac{1}{2}\arctan \left( { - \frac{{2\tau_{xy} }}{{\sigma_{x} - \sigma_{y} }}} \right) $$

The initial fracture collapse angle *θ*_1_ and the periodic fracture collapse angle *θ*_2_ of the bedrock are determined from Eqs. (1), (3), and (4) as follows,

5 $$ \left\{ {\begin{array}{*{20}l} {\theta_{1} = \frac{\pi }{2} - \frac{1}{2}\arctan \left[ {\frac{{\frac{12x}{{h^{3} }}\left( {\frac{{h^{2} }}{4} - y^{2} } \right)}}{{\frac{{6yL^{2} }}{{h^{3} }} - \frac{{6x^{2} y}}{{h^{3} }} + \frac{{6y^{3} }}{{h^{3} }} - \frac{21y}{{10h}} + \frac{1}{2}}}} \right]} \hfill \\ {\theta_{2} = \frac{\pi }{2} - \frac{1}{2}\arctan \left[ {\frac{{\frac{12x}{{h^{3} }}\left( {\frac{{h^{2} }}{4} - y^{2} } \right)}}{{ - \frac{{6x^{2} y}}{{h^{3} }} + \frac{{6y^{3} }}{{h^{3} }} - \frac{21y}{{10h}} + \frac{1}{2}}}} \right]} \hfill \\ \end{array} } \right. $$

Mechanical analysis indicates that the initial fracture collapse angle is greater than that of the periodic fracture collapse. Consequently, the bedrock fractures induced by coal layer mining will be asymmetric, as will the movement of the overlying thick loose layers. The resulting surface subsidence under the thick loose strata will also deviate from the mining center, leading to asymmetric subsidence.

### Asymmetric analogous hyperbola model of overburden movement

#### Analogous hyperbola model of overburden movement

Historically, research on rock stratum movement and mining-induced surface subsidence has been fragmented. The movement of rock layers has predominantly been analyzed using rock fracture mechanics theory, suggesting that as coal resources are extracted, the overlying rock layers exhibit a stair-step fracture pattern^15,16^. Concurrently, the surface subsidence caused by mining assumes an “inverted funnel” shape.

**Figure 3:**
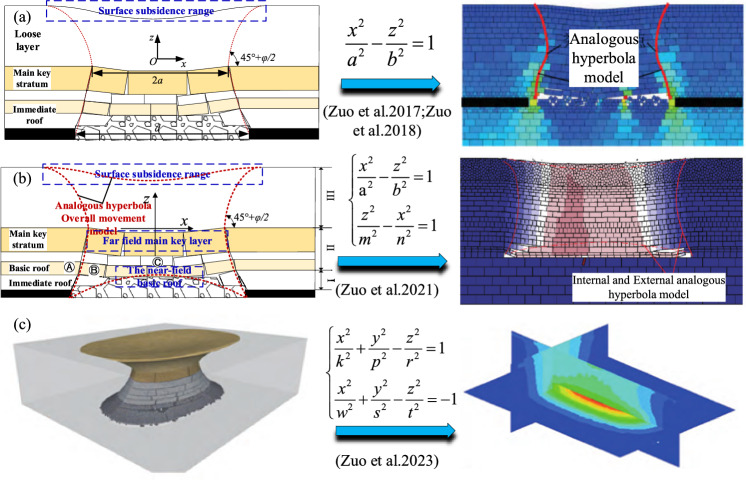
Analogous hyperbola movement model of rock stratum. (**a**) Analogous hyperbola model; (**b**) conjugate internal and external analogous hyperbola model; (**c**) spatial “single-leaf hyper-boloid” model.

Building on key strata theory, our team proposed that the surface subsidence curve for thick loose layers is concave. The collapse zone above the mined-out area is convex, approximating symmetry around the main key stratum. We also analyzed the mechanics of caving and fracture arches, elucidating the characteristics of the inverted funnel arch within the fracture zone. This research led to the development of the analogous hyperbola model for overburden movement and the conjugate internal and external analogous hyperbola model for rock stratum movement and surface subsidence^27^. Ultimately, we extended the two-dimensional model to three-dimensional space, introducing a spatial “hyperbolic surface” movement model^24^, as detailed in Fig. 3. Specifically, the external analogous hyperbola model for rock stratum movement is expressed by Eq. (6).

6 $$ \frac{{x^{2} }}{{a^{2} }} - \frac{{z^{2} }}{{b^{2} }} = 1 $$

Parameters *a* and *b* can be expressed as Eqs. (7) and (8).

7 $$ a = \left( {d - \mathop \sum \limits_{j = 1}^{i} \cot \theta_{1j} h_{j} - \mathop \sum \limits_{j = 1}^{i} \cot \theta_{2j} h_{j} } \right)/2 $$

8 $$ b = {\raise0.7ex\hbox{${aH}$} \!\mathord{\left/ {\vphantom {{aH} {\sqrt {\left[ {a + \smallint_{0}^{H} \cot \left( {45^{ \circ } + \varphi /2} \right){\text{d}}H} \right]^{2} - a^{2} } }}}\right.\kern-0pt} \!\lower0.7ex\hbox{${\sqrt {\left[ {a + \smallint_{0}^{H} \cot \left( {45^{ \circ } + \varphi /2} \right){\text{d}}H} \right]^{2} - a^{2} } }$}} $$

where *d* is the mining length of the coal seam, m; *I* is the number of rock strata. *θ*_1j_,*θ*_2j_ is the initial and periodic collapse angles of the rock strata, °. *H* is the depth of the loose layer from the top of the bedrock, m. *h*_j_ is the thickness of each rock layer, m.*φ* is the internal friction angle of the loose layer, °.

Substituting (*D*/2, *H*) into Eq. (6), the range of surface subsidence D can be obtained, as shown in Eq. (9).

9 $$ D = 2a\sqrt {1 + \frac{{H^{2} }}{{b_{{}}^{2} }}} $$

For a more comprehensive depiction of the relationship between surface subsidence and rock stratum movement, the expression for the internal analogous hyperbola model governing the movement of rock strata and surface subsidence during horizontal coal seam mining in thick loose layers is presented in Eq. (10).

10 $$ \frac{{z^{2} }}{{m^{2} }} - \frac{{x^{2} }}{{n^{2} }} = 1 $$

By substituting $\left( {a + \smallint_{0}^{H} \cot \left( {45^{ \circ } + \frac{\varphi }{2}} \right){\text{d}}H,\;H_{{\text{t}}} /{2}} \right)$ and (*d*/2, -*H*_t_/2) into Eq. (3), the physical parameters *m* and *n* for the internal analogous hyperbola model can be determined as,

11 $$ m = \sqrt {\frac{{\frac{1}{4}d^{2} H_{{\text{t}}}^{2} - H_{{\text{t}}}^{2} \left[ {a + \smallint_{0}^{H} \cot \left( {45^{ \circ } + \frac{\varphi }{2}} \right){\text{d}}H} \right]^{2} }}{{d^{2} - 4\left[ {a + \smallint_{0}^{H} \cot \left( {45^{ \circ } + \frac{\varphi }{2}} \right){\text{d}}H} \right]^{2} }}} $$

12 $$ n = \frac{Dm}{{2\sqrt {\frac{1}{4}H_{{\text{t}}}^{2} - m^{2} } }} $$

where *H*_t_ represents the depth of the coal seam, $H_{{\text{t}}} = H + \mathop \sum \limits_{i = 1}^{m} h_{i}$.

Equations (6) and (10) are collectively referred to as the overall model of the internal and external analogous hyperbola movement of rock strata. The maximum value of surface subsidence, *H*_1_, and the expression for the maximum value of surface subsidence movement is* H*_1_ = *H*_t_/2-*m*.

#### Asymmetric analogous hyperbola model of overburden movement

**Figure 4:**
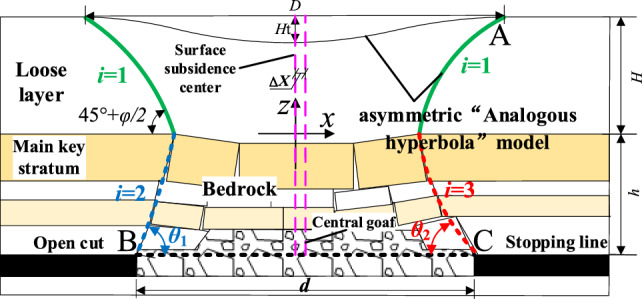
The internal and external asymmetric analogous hyperbola model of overlying strata.

Previous research has contributed to the development of a unified theory for mining-induced overburden movement and surface subsidence. However, practical engineering experiences and insights from the mechanics of asymmetric fractures in mined rock strata suggest that both bedrock fractures and loose layer movements display asymmetric changes during coal mining beneath thick loose layers. Further investigation into the impact of asymmetric bedrock fractures on overlying loose layers reveals from the above mechanical analysis of initial and periodic fractures in rock layers that mining affects the open-cut side more significantly than the stop-line side during coal mining. This discrepancy in initial and periodic collapse angles results in asymmetric movement of both bedrock and loose layers on the open cut and stop line sides. Therefore, utilizing the internal and external analogous hyperbolic model of overburden movement, and taking into account the influence of varied collapse angles that induce asymmetric bedrock collapse and movement of both loose layers and bedrock, hyperbolic equations are formulated by introducing different virtual semi-axis lengths b. Here, the x-axis aligns with the orientation of the main key stratum, and the z-axis is normal to the midpoint of the main key stratum fracture, as depicted in Fig. 4.

Substituting point A: $\left( {a + \smallint_{0}^{H} \cot \left( {45^{ \circ } + \varphi /2} \right){\text{d}}H,H} \right)$ from Fig. 4 into Eq. (6) yields the movement curve of the upper loose layer of the bedrock, where '*a*' is half the length of the fracture in the key stratum; By substituting point B: $\left( { - a - \mathop \sum \limits_{j = 1}^{i} \cot \theta_{1j} h_{j} , - \mathop \sum \limits_{j = 1}^{i} h_{j} } \right)$ into Eq. (1), the movement curve of the bedrock below the loose layer and above the open cut can be derived; Similarly, substituting point C: $\left( {a + \mathop \sum \limits_{j = 1}^{i} \cot \theta_{2j} h_{j} , - \mathop \sum \limits_{j = 1}^{i} h_{j} } \right)$ into Eq. (6) yields the movement curve of the bedrock below the loose layer and above the stop line.

In summary, the external asymmetric analogous hyperbola model describing overburden movement beneath thick loose layers is as follows:

13 $$ \frac{{x^{2} }}{{a^{2} }} - \frac{{z^{2} }}{{b_{i}^{2} }} = 1 $$

where *b*_*i*_ is the virtual semi-axis length of the hyperbolic model, where *i* = 1, 2, and 3, for *i* = 1, the virtual semi-axis length of the movement curve for the upper loose layer of the bedrock (where *z* > 0) is $b_{1} = aH/\sqrt {\left[ {a + \smallint_{0}^{H} \cot \left( {45^{ \circ } + \varphi /2} \right){\text{d}}H} \right]^{2} - a^{2} }$; for *i* = 2, the virtual semi-axis length of the bedrock movement curve on the open cut side (where x ≤ 0, z ≤ 0) is $b_{2} = a\mathop \sum \limits_{j = 1}^{i} h_{j} /\sqrt {\mathop \sum \limits_{j = 1}^{i} \cot \theta_{1j} h_{j} \left( {\mathop \sum \limits_{j = 1}^{i} \cot \theta_{1j} h_{j} + 2a} \right)}$; for *i* = 3, the virtual semi-axis length of the bedrock movement curve on the stop line side (where x > 0, z ≤ 0) is $b_{3} = a\mathop \sum \limits_{j = 1}^{i} h_{j} /\sqrt {\mathop \sum \limits_{j = 1}^{i} \cot \theta_{2j} h_{j} \left( {\mathop \sum \limits_{j = 1}^{i} \cot \theta_{2j} h_{j} + 2a} \right)}$.

Based on the above formulas, it is apparent that the centre of subsidence beneath the loose layer surface corresponds to the centre of the key stratum, but it does not align with the centre of the mined-out area. We define the difference between these two centres as the degree of offset of the surface subsidence centre, Δ*X*, as illustrated in Eq. (14).

14 $$ \Delta X = \frac{d}{2} - \sum\limits_{{j{ = 1}}}^{i} {\cot \theta_{1j} } h_{j} - a $$

The symbols in the equation remain as previously defined. The collapse angle *θ*_1*j*_ can be deduced from the known value of Δ*X* and other parameters. The magnitude of Δ*X* can be used to characterize the degree of offset in the curve representing overburden movement.

The asymmetric analogous hyperbola model describing overburden movement beneath thick loose layers is as follows:

15 $$ \frac{{z^{2} }}{{m^{2} }} - \frac{{\left( {x + \Delta X} \right)^{2} }}{{n^{2} }} = 1 $$

The asymmetric analogous hyperbola model introduces asymmetry, reflecting the reality that the initial and periodic collapses of rock layers caused by geological characteristics and mining operations are not symmetrical, with fractures and displacements often being uneven. This model integrates detailed mechanical analysis with empirical observations, bridging the gap between theoretical predictions and practical observations. This model can more accurately predict fracture patterns and strata movements, providing reliable guidance for engineering decisions.

#### Sensitivity analysis of model parameters

##### Impact of loose layer thickness on overburden movement morphology

**Figure 5:**
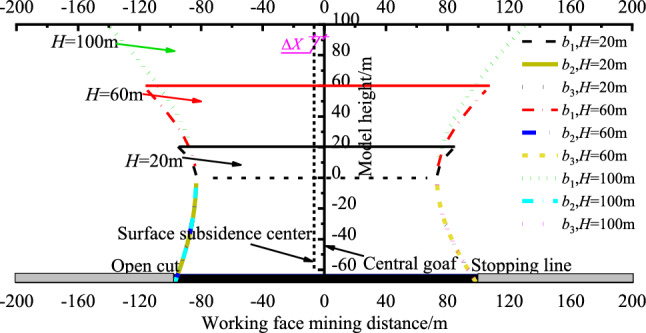
Relationship between overburden migration form and loose layer thickness *H.*

To investigate the impact of loose layer thickness *H* on the morphology of overburden movement, we considered a working face length of *d* = 195 m, collapse angles of *θ*_1j_ = 78.6° on the open cut side and *θ*_2j_ = 69.7° on the stop line side, an internal friction angle of *φ* = 30° in the loose layer, and a bedrock layer thickness of *h* = 67.5 m. We varied the loose layer thickness *H* to 20, 60, and 100 m and analyzed its relationship with the morphology of overburden movement, illustrated in Fig. 5.

Figure 5 shows that the overburden movement morphology resulting from coal mining under thick loose layers is analogous to hyperbola and asymmetrical about the centre of the mined-out area, with a surface subsidence centre offset value Δ*X* of 6.4 m. With the thickness *H* of the loose layer increasing from 20 to 100 m, the virtual semi-axis *b*_1_ values of the loose layer are 35.5, 57.7, and 70.3, while the virtual semi-axis *b*_2_ and *b*_3_ values of the bedrock layer remain constant at 109.7 and 78.4. This suggests that as the virtual semi-axis *b*_1_ value of the loose layer increases, the movement curve of the loose layer becomes steeper, and the angle it forms with the horizontal plane also increases. Consequently, a greater thickness of the loose layer corresponds to a larger extent of subsidence at the surface of the loose layer. The model curves may be related to the thickness and physical properties of the loose layer. With varying physical properties of the overlying loose layer, the curves and results can exhibit significant differences. Additionally, the position of the main key strata plays a role. The results are applicable to similar geological conditions where the main key strata are overlain by loose layers.

##### Impact of bedrock thickness on overburden movement morphology

**Figure 6:**
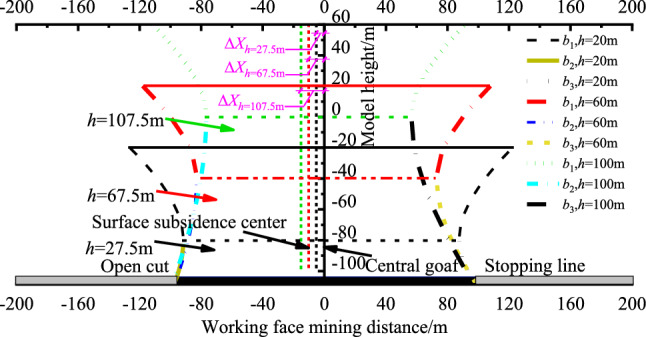
Relationship between overburden migration and bedrock thickness *h.*

For the investigation of how bedrock thickness h influences the morphology of overburden movement, with a fixed loose layer thickness H = 60 m, we varied the bedrock layer thickness to *h* = 27.5, 67.5, and 107.5 m. Calculations were conducted to analyze how different bedrock thicknesses *h* relate to the morphology of overburden movement, as depicted in Fig. 6.

Figure 6 shows that as the bedrock layer thickness h gradually increases from 20 to 100 m, the surface subsidence centre offset values Δ*X* increase to 2.1 m, 6.4 m, and 10.7 m respectively. This suggests that as the bedrock layer thickness increases, the surface subsidence centre gradually shifts towards the open cut side, accompanied by an increase in the degree of surface subsidence centre offset. In these cases, the virtual semi-axis *b*_2_ values of the bedrock layer are − 77.0, − 109.7, and − 123.8, and the b3 values are − 56.2, − 78.4, and − 86.5. Simultaneously, the virtual semi-axis *b*_1_ values are 62.5, 57.7, and 52.5, suggesting that as the thickness of the bedrock layer increases, the angle between the loose layer movement curve and the horizontal line decreases, resulting in a reduced subsidence range for the loose layer. The surface subsidence centre offset value remains consistent with the increase in loose layer thickness. A thicker loose layer corresponds to a greater virtual semi-axis length *b*_1_, resulting in a steeper curve for loose layer movement and an increased surface subsidence range. Conversely, an increase in bedrock layer thickness leads to a larger surface subsidence centre offset value. A smaller hyperbolic virtual semi-axis length *b* implies a smaller angle between the loose layer movement curve and the horizontal line, leading to a flatter curve for rock layer movement and a reduced surface subsidence range. The theoretical predictions of the model for surface subsidence are more suitable under conditions of full mining extraction. Before the bedrock layer has fully collapsed, surface subsidence is relatively minor. Therefore, there may be certain inaccuracies in the prediction results for non-fully mined conditions.

#### A similar model experiment for mining in thick loose layers

##### Design of a similar model experiment

**Figure 7:**
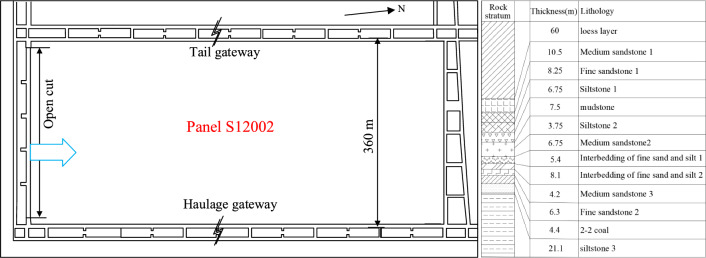
S12002 working face layout.

**Figure 8:**
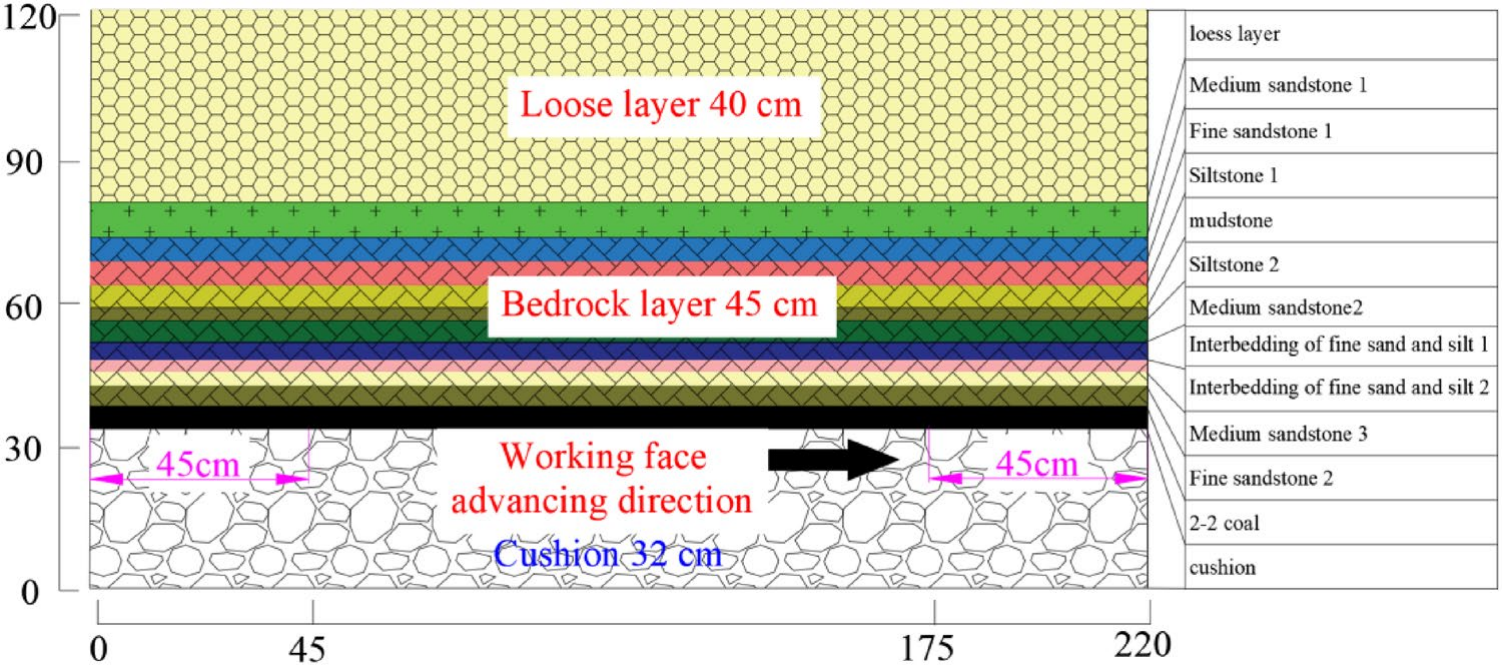
Experimental model of similar materials.

To validate the rationale behind the asymmetric fracture mechanics analysis of rock layers mentioned earlier, a corresponding model experiment was conducted to simulate mining operations in thick loose layers. The experiment aimed to study the movement and destruction characteristics of the overlying bedrock and thick loose layers during coal mining. The S12002 working face of Ningtiaota mine is located south of the main south wing tunnel in the mining area, featuring a coal seam thickness ranging from 4 to 4.8 m and an inclination angle of less than 2°, suggesting almost horizontal mining of the coal seam. The layout of the working face is depicted in Fig. 7. The similar material model experiment was designed, as shown in Fig. 8. The stratigraphic rock parameters and similar material proportions are presented in Table 1.

**Table 1:** Physical and mechanical parameters.

No	Thickness (m)	Model layer thickness (cm)	Compressive strength (MPa)	Density (kg/m^3^)	Elastic modulus (GPa)	Cohesion (MPa)	Internal friction angle (°)	Poisson ratio	Ratio number: (aeolian sand: lime: gypsum)	Lithology
1	60	40	0.29	1840	0.03	0.08	30	0.3	–	Loess layer
2	10.5	7	32.4	2334	5.87	8.02	37.56	0.33	373	Medium sandstone 1
3	8.25	5.5	24.12	2139	3.36	8.25	28.86	0.3	555	Fine sandstone 1
4	6.75	4.5	37.78	2440	3.68	14.1	23.67	0.29	464	Siltstone 1
5	7.5	5	46.39	2451	4.67	17.7	20.68	0.22	455	Mudstone
6	3.75	2.5	46	2442	5.44	13.6	24.11	0.25	455	Siltstone 2
7	6.75	4.5	30.87	2334	5.87	8.02	37.56	0.33	473	Medium sandstone2
8	5.4	3.6	50.22	2460	6.79	13.77	37.57	0.23	455	Interbedding of fine sand and silt 1
9	8.1	5.4	62.55	2394	1.2	18.3	34.17	0.27	355	Interbedding of fine sand and silt 2
10	4.2	2.8	45.15	2123	10.9	10.2	40.73	0.34	455	Medium sandstone 3
11	6.3	4.2	72.91	2328	11.2	20.3	31.56	0.25	346	Fine sandstone 2
12	4.4	2.93	13.8	1540	1.1	1.3	28	0.36	573	2–2 coal
13	21.1	14.07	20.5	2306	11.2	10.2	40.73	0.34	564	Siltstone 3
14	27	18	39.1	2106	11.3	20.3	31.56	0.25	464	Siltstone 4

**Figure 9:**
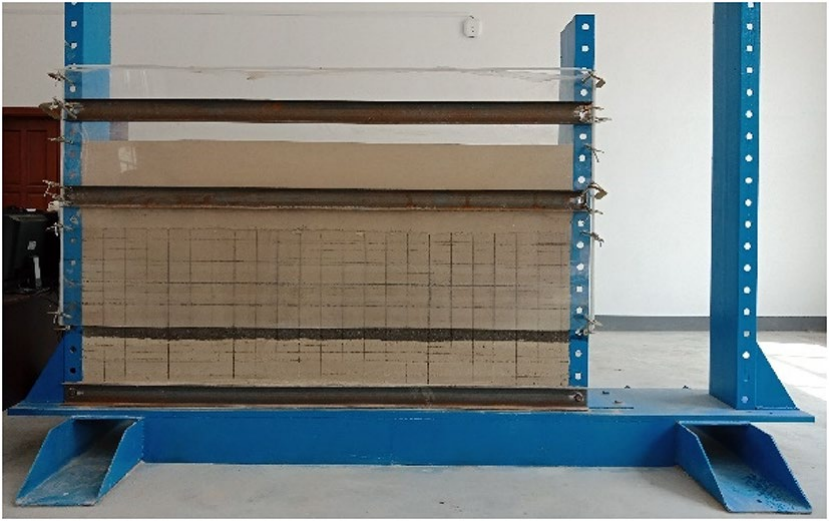
Similar simulation test bench.

The experiment employed a self-developed similar material simulation platform, with the model dimensions of 2.2 m in length, 0.3 m in width, and a maximum simulation height of 2.0 m, as shown in Fig. 9. The geometric similarity ratio (*α*_L_) of the similar material model experiment is 150, the time similarity ratio (*α*_t_) is 12.25, the bulk density similarity ratio (*α*_γ_) is 1.56, and the stress similarity ratio(*α*_F_) is 234. The materials used in the similar material simulation experiment include aeolian sand, lime, gypsum, and water, with mica sheets used for layering. The material proportions for laying are shown in Table 1. Firstly, the similar materials are weighed and measured according to the order and ratio of rock strata from bottom to top, and poured into the mixer to add water to stir evenly. Then, the mixed ratio materials are placed at the bottom of the test bench to consolidate and level off, and mica is placed in layers. Laying to a certain height and then heightening the U-shaped steel baffle layer by layer until the model laying is completed. The experimental model is shown in Fig. 9. After the similar model is dried and formed, the baffles on both sides are removed, the measuring points are set on the surface of the model, the camera is debugged, and the coal seam mining work is carried out within 1–2 days. An open cut was set at 45 cm from the left side of the model, with a simulated coal seam excavation speed of 5 cm/h. Mining commenced 45 cm from the left boundary of the model and concluded 45 cm from the right boundary.

##### Analysis of overburden movement rules

**Figure 10:**
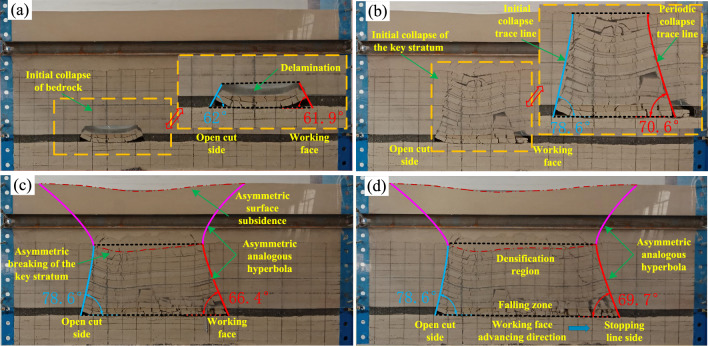
The overburden caving form during coal mining. (**a**) 40 cm; (**b**) 65 cm; (**c**) 100 cm; and (**d**) 130 cm.

Figure 10 shows the results of a similar model experiment on overburden collapse morphology during the mining process.

Figure 10 shows that when the mining face is excavated to 40 cm, the roof reaches its ultimate span, resulting in the initial fracture. The collapse angle on the open cut side of the overlying rock is 62°, whereas on the working face side, it measures 61.9°. At 65 cm, the upper bedrock layer undergoes initial fracture, and the loose layer experiences minor localized subsidence, reaching its peak deformation. There is localized collapse on the working face side, characterized by a non-isosceles trapezoid shape. The collapse angle formed on the open cut side of the overlying rock is 78.6°, while on the working face side, it is 70.6°. At 65 cm, it enters a cyclic failure stage. At 100 cm, the subsidence deformation in the loose layer increases. The collapse angle formed on the open cut side of the overlying rock is 78.6°, while on the working face side, it is 66.4°. The collapse of the key stratum shows an obvious asymmetric fracture, leading to asymmetric surface subsidence and the initial formation of the asymmetric analogous hyperbole characteristic of the overlying rock layer movement. At 130 cm, the surface subsidence reaches its maximum depth. The collapse angle on the open cut side of the overlying rock is 78.6°, while above the stopping line, it is 69.7°. Overall, the movement of the overlying rock displays a clear characteristic of asymmetric analogous hyperbolic movement.

**Figure 11:**
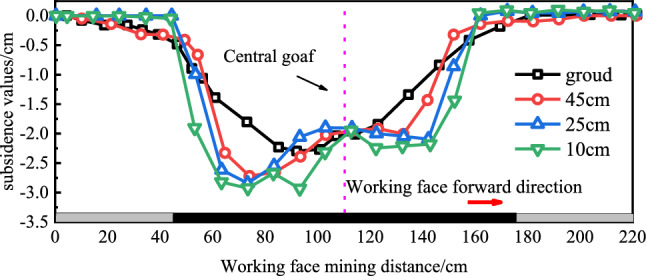
Settlement displacement curves of overburden rock.

During excavation, monitoring at measurement points yielded subsidence curves for various overlying strata, as illustrated in Fig. 11. The regions 10 cm and 25 cm above the coal seam represent bedrock layers, and 45 cm is the upper part of the key stratum.

Figure 11 shows that the maximum subsidence at 10 cm above the coal seam is 2.9 cm, at 25 cm above, it is 2.8 cm, and at 45 cm above, it is 2.7 cm. The intermittent fractures in the rock layers during periodic bedrock rupture cause gradual compaction of central layers in the goaf, resulting in increased subsidence on both sides of the collapse. The maximum surface subsidence is 2.3 cm. This indicates that with the advancement of the working face, the subsidence values on the open-cut side of the rock layer are greater than on the other side. The center of surface subsidence is not at the central goaf, and the subsidence curve of the overlying strata shows asymmetry, centered around the middle of the mining face.

#### Overlying strata movement discrete element numerical simulation

##### Numerical model

**Figure 12:**
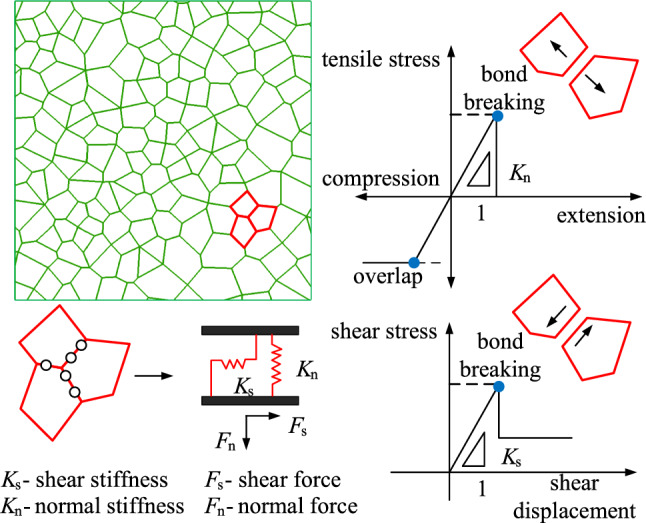
Microscopic characteristics and constitutive contact behavior of Voronoi diagram.

The discrete element program UDEC is particularly suitable for simulating blocky systems with large movements and large deformations in discontinuous media. Rocks are considered brittle materials with tensile strength less than compressive strength. Tensile strength plays a crucial role in rock failure, thus a Mohr–Coulomb elastoplastic constitutive model that considers tensile failure is chosen. By using the Voronoi rock blocks generated in UDEC, as shown in Fig. 12, it is possible to cement the polygons and rock particles together using rigid or deformable polygonal blocks to achieve an effect similar to the natural failure process. When simulating the surface loose layer in underground mining, using UDEC-DM has significant advantages and helps in understanding the impact of underground mining on the surface loose layer^29,30^^.^ The difference in stiffness and size between block material and joint material may cause UDEC to take a long time to converge during calculations. To prevent virtual joint movement, high stiffness can be assigned to the joints, but it is important not to assign excessively high stiffness values. A common rule of thumb is to set the joint stiffness to ten times the highest stiffness of the adjacent elements.

**Figure 13:**
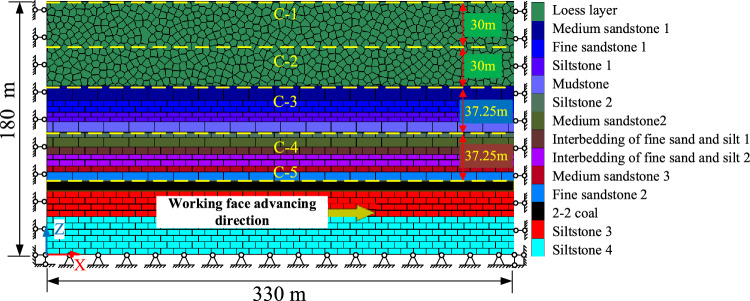
Model unit division and boundary setting.

The numerical model of S12002 working face in Ningtiaota mine was established using UDEC 7.0. The aim is to validate the proposed asymmetric analogous hyperbola model of overlying strata movement. The numerical model is 330 m long, and 180 m high, with a coal seam thickness of 4.4 m and a primary key stratum thickness of 10.5 m. The primary key stratum is positioned beneath the loose layer, at the top of the bedrock, 64.5 m from the coal seam bottom, as depicted in Fig. 13. The X-axis aligns with the working face, the Z-axis represents the vertical direction, and gravity acts on the entire model. The model's left, right, and bottom sides are fixed, while the top boundary is free, with a side pressure coefficient set to *λ* = 1. The loose layer was divided into Voronoi blocks, and the bedrock joints were divided into rectangular blocks, resulting in a total of 21,383 elements. To minimize boundary effects, mining initiates at 67.5 m from the left model boundary and concludes at 100 m from the right model boundary. The physical and mechanical parameters for rock layers are detailed in Table 1.

The selected rock failure criterion is the Mohr–Coulomb criterion. The loose layer thickness is varied at *H* = 20, 60, and 100 m, and the bedrock layer thickness at *h* = 27.5, 67.5, and 107.5 m, with mining progressing by 5 m increments. Horizontal measuring lines C-1 to C-5 are positioned at distances above the coal seam (15, 37.5, 67.5, 97.5, 127.5 m). At 67.5 m above the coal seam lies the top of the bedrock layer, at 97.5 m is the middle of the thick loose layer, and at 127.5 m is the surface level.

## Simulation results analysis

### Vertical movement characteristics of overlying strata

**Figure 14:**
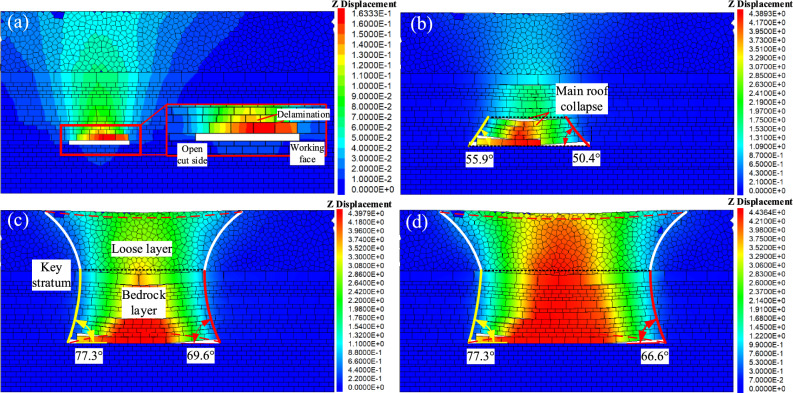
Vertical displacement diagram of overburden rock. (**a**) 60 m; (**b**) 120 m; (**c**)150 m; and (**d**). Reaches 195 m.

Figure 14 depicts cloud maps of vertical displacement of overlying strata at different mining lengths.

In Fig. 14a, it is evident that when the mining face reaches 60 m, the immediate roof remains unbroken, causing only localized minor subsidence. At 120 m (Fig. 14b), damage to the overlying strata propagates to the old roof, where the initial collapse takes on a funnel-shaped pattern. Small-scale subsidence occurs in the key stratum and at the surface. The collapse angle is 55.9° on the open cut side and 50.4° on the working face side. At 150 m (Fig. 14c), the lower part of the key stratum exhibits a non-isosceles trapezoidal collapse, while the upper loose layer displays funnel-shaped damage. The initial formation of goaf rock layer movement and surface subsidence manifests an asymmetric analogous hyperbola characteristic. The collapse angle is 77.3° on the open cut side and 69.6° on the working face side. At 195 m (Fig. 14d), with the continued advancement of the working face, strata closure occurs, and the height of overlying strata dam-age ceases to increase. Surface subsidence tends towards stability, and the overlying strata experience periodic collapse. The collapse angle is 77.3° on the open cut side and 66.6° on the working face side, indicating a more pronounced asymmetric analogous hyperbolic characteristic.

**Figure 15:**
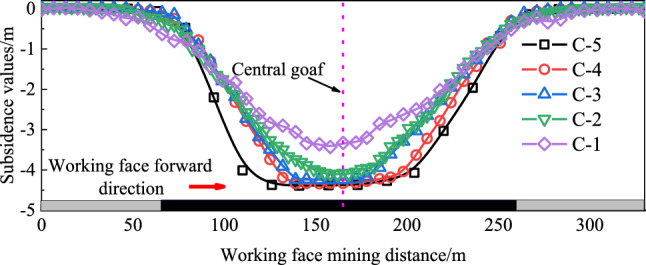
Vertical displacement curves with a mining length of 195 m.

Figure 15 shows the vertical displacement curves for the upper five measuring lines of overlying strata when the coal seam is mined to a depth of 195 m.

Figure 15 shows that the subsidence curve is not entirely symmetric about the centre of the goaf. The discontinuous fracture resulting from the initial and periodic collapse of overlying strata leads to a slightly greater impact on the open-cut side compared to the working face side. Line C-5 experiences a maximum subsidence of 4.4 m due to trapezoidal fracturing of the bedrock layer, severe fragmentation in the lower part of the collapse zone, and compaction of fragmented rocks by the upper part of the collapse zone, resulting in significant subsidence. Line C-4 experiences 4.3 m of subsidence due to gradual compaction of fissures in the away-from-face zone, leading to maximum subsidence. At line C-3, subsidence reaches 4.2 m due to the coal seam's extensive extraction, stabilizing the subsidence. Line C-2 experiences 4.1 m subsidence, while line C-1 experiences 3.4 m subsidence. In summary, during excavation, the subsidence tends to favour the open cut side, eventually impacting the surface of the loose layer, creating an asymmetric overlying strata movement characteristic in the goaf area.

### Morphological variations in overlying strata movement with different thicknesses of the loose layer

**Figure 16:**
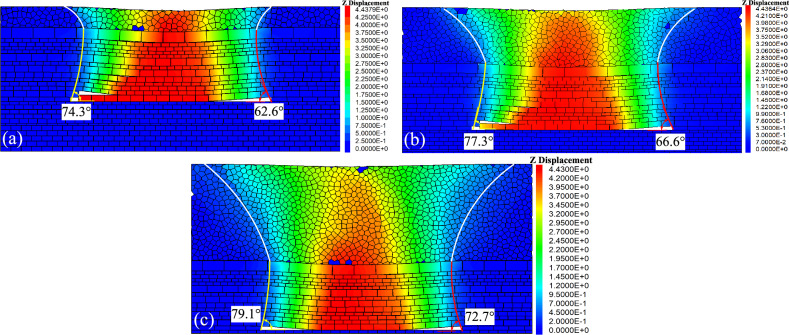
Vertical displacement with different loose layer height. (**a**) *H* = 20 m; (**b**) *H* = 60 m; and (**c**) *H* = 100 m.

Figure 16 illustrates cloud maps of vertical displacement at different heights of the loose layer.

Figure 16 shows that the asymmetric movement of overlying strata demonstrates distinct asymmetry under different heights of the loose layer (*H*). With a constant height of the bedrock layer (*h*), at *H* = 20 m, the initial collapse angle is 74.3°, and the periodic collapse angle is 62.6°. At *H* = 60 m, the initial collapse angle is 77.3°, and the periodic collapse angle is 66.6°. At *H* = 100 m, the initial collapse angle is 79.1°, and the periodic collapse angle is 72.7°. Through measurements, the displacement value Δ*X* of the subsidence centre from the surface is approximately 10 m. As the loose layer height increases, the collapse angle enlarges, Δ*X* undergoes minimal changes, the collapse length of the key stratum increases, and the range of surface subsidence gradually expands.

### Morphological variations in overlying strata movement with different heights of the bedrock layer

**Figure 17:**
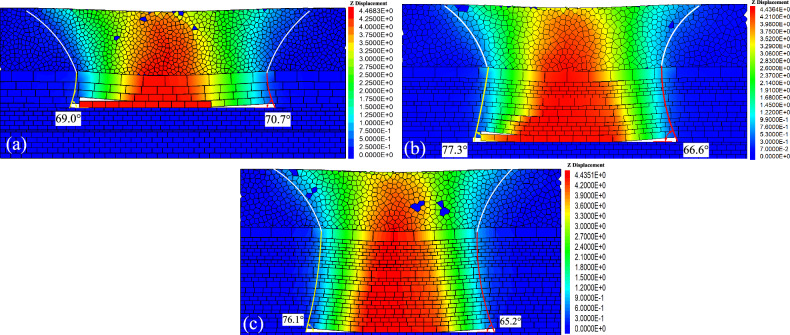
Vertical displacement with different bedrock height. (**a**) *h* = 27.5 m; (**b**) *h* = 67.5 m; and (**c**) *h* = 107.5 m.

Figure 17 illustrates cloud maps of vertical displacement at different heights of the bedrock layer.

Figure 17 shows that the initial collapse angle is 78.0°, and the periodic collapse angle is 67.6° at *h* = 27.5 m with a constant of *H* value, The displacement value Δ*X* of the subsidence centre from the surface is approximately 5 m. At *h* = 67.5 m, the initial collapse angle is 77.3°, and the periodic collapse angle is 66.6°, with Δ*X* at about 10 m. With the bedrock layer height is 107.5 m, the initial collapse angle decreases to 76.1°, and the periodic collapse angle decreases to 65.2°, with Δ*X* approximately 13 m. As the bedrock layer height increases, the collapse angle decreases, Δ*X* increases, the collapse length of the key stratum diminishes, and the range of surface subsidence gradually decreases.

## Discussion

### Discussion of model results

To validate the effectiveness of the proposed asymmetric analogous hyperbola model for overlying strata movement with varying collapse angles in a thick loose layer, a comparative analysis is performed among theoretical computational results, analogous model test outcomes, and numerical simulation results.

For excavation lengths (*d*) of 150 m and 195 m, with a loose layer thickness of *h*_1_ = 60 m, bedrock thickness of *h*_2_ = 67.5 m, and an internal friction angle *φ* = 30° within the loose layer, incorporating these parameters into the model yields the following results: at *d* = 150 m, the outer curve values are *a*_1_ ≈ 55.7, *b*_11_ ≈ 47.0, *b*_12_ ≈ 91.2, *b*_13_ ≈ 78.4; inner curve values are *m*_1_ ≈ 61.5; *n*_1_ ≈ 334.9. At *d* = 195 m, the outer curve values are *a*_2_ ≈ 78.2, *b*_21_ ≈ 57.7, *b*_22_ ≈ 109.7, *b*_23_ ≈ 78.4; inner curve values are *m*_2_ = 60.6; *n*_2_ = 342.6. The surface subsidence ranges are approximately *D*_150_ ≈ 180.7 ≈ 180.7 m for 150 m excavation and *D*_195_ ≈ 225.7 m for 195 m excavation, with subsidence values of *H*_150_ = 2.2 m and *H*_195_ = 3.2 m, respectively.

**Figure 18:**
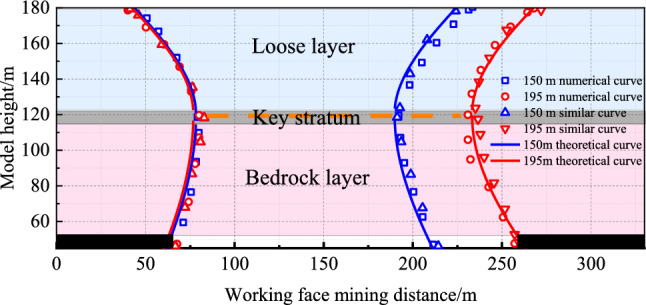
Overburden rock movement curves.

**Figure 19:**
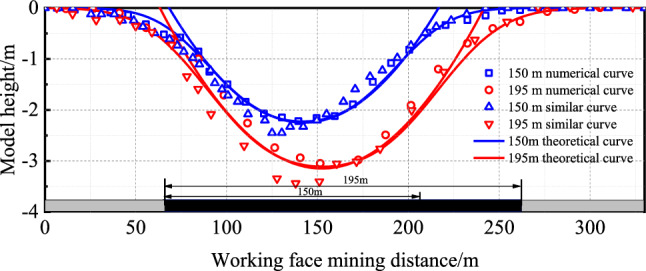
Surface subsidence curve.

A comparison of the theoretical outcomes with numerical simulation and analogous model test results is depicted in Figs. 18 and 19.

Figure 18 shows that the movement pattern of overlying strata exhibits an asymmetric analogous hyperbola shape during excavation beneath a thick loose layer. The movement curve of the loose layer appears trapezoidal, while that of the bedrock shows a rectangular shape. As the excavation length increases, the range of surface subsidence expands. At *d* = 150 m, the theoretical surface subsidence range is 180.7 m; the analogous model test yields 187.6 m, exceeding the theoretical value by 3.8%. The numerical simulation results in 195.6 m, surpassing the theoretical value by 8.2%. At *d* = 195 m, the theoretical surface subsidence range is 225.7 m; the analogous model test results in 233.5 m, exceeding the theoretical value by 3.5%. The numerical simulation produces 237.9 m, surpassing the theoretical value by 5.4%.

Figure 19 shows that the surface subsidence curve takes on the shape of an “inverted arch,” where the arch depth represents the surface subsidence value, and the arch width denotes the subsidence range. The maximum subsidence of the overlying strata in the goaf area is not exactly at the centre of the goaf and shifts with the excavation progress. At *d* = 150 m, the theoretical surface subsidence is 2.3 m; in the analogous model test, it measures 2.2 m, exceeding the theoretical value by 6.5%. The numerical simulation indicates 2.45 m, which is 4.5% less than the theoretical value. At *d* = 195 m, the theoretical surface subsidence is 3.2 m; the analogous model test records 3.44 m, surpassing the theoretical value by 7.5%. Meanwhile, the numerical simulation results in 3.05 m, which is 4.9% less than the theoretical value. It is evident that the rock strata movement exhibits an asymmetric analogous hyperbola characteristic, and the discrepancies among the three measurements are relatively small.

### Model verification through field measurements

**Figure 20:**
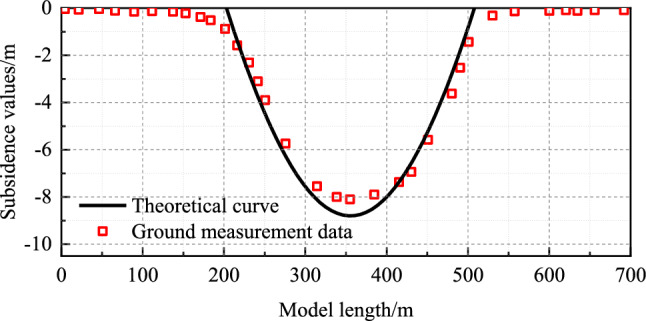
The surface subsidence curve of Baodian 1312 working face^27^.

Figure 20 illustrates the comparative validation of surface subsidence data from the Baodian 1312 working face^31^ with the asymmetric analogous hyperbola model of overlying strata movement.

The collapse angles are approximately 77° on the open cut side and around 67° on the stoping line side, with an internal friction of 22° within the loose layer. Applying these parameters to the model yields obtained values of *m* = 170 and *n* = 669. The measured surface subsidence value is *H* = 8.1 m, whereas the theoretical value is *H* = 8.8 m, resulting in an error of approximately 8%. The subsidence centre Δ*X* = 17.3 m is biased towards the open-cut side.

In summary, the theoretical predictions of the asymmetric analogous hyperbola model closely match other calculated results, showing minimal errors. This suggests that the asymmetric analogous hyperbola model is proficient in predicting the movement of overlying strata and surface subsidence patterns in thick loose coal layers. Future research should focus on refining the model to incorporate more complex geological variables and mining conditions. Additionally, extending the model’s application to other types of mining and geological disturbances will enhance its utility. Development of integrated tools and software based on the model can further aid in real-time subsidence monitoring and prediction, improving mine safety and environmental protection measures.

## Conclusion

This study addresses the overlying strata movement and surface subsidence resulting from coal mining be-neath thick loose layers. The asymmetric analogous hyperbola model for overlying strata movement was estab-lished by considering the asymmetrical collapse due to different initial and periodic collapse angles (θ1 and θ2, respectively). The effectiveness of the model was verified through similar model experiments and numerical simulation experiments. The main conclusions are as follows:

1. An analogous hyperbola model for the asymmetric movement of overlying strata was established, which considers the asymmetrical collapse due to different initial and periodic collapse angles.
2. The model demonstrated that the displacement of the subsidence centre (Δ*X*) increases with the thickness of the bedrock layer, while the length of the hyperbolic curve's imaginary semi-axis (*b*) decreases, reducing the surface subsidence range. Conversely, the loose layer thickness has minimal impact on Δ*X* but increases the surface subsidence range.
3. Similarity model tests and numerical simulations confirmed the model's predictions, with a maximum relative error of less than 7.5% compared to theoretical calculations and an 8% discrepancy with real-world data from the Baodian 1312 working face.

The above results enhance the capability of the established asymmetric analogous hyperbola model to effec-tively depict the curves of overlying strata movement, as well as the depth and range of surface subsidence occur-ring due to coal mining under thick loose layers. The research results will provide theoretical support for the accurate prediction of overburden fracture movement and surface subsidence and disaster prevention and control under the condition of thick loose layer mining.

## Data Availability

All data, models, and code generated or used during the study appear in the submitted article.
